# Schmerzen, Schmerzmitteleinnahme und mentale Gesundheit von Intensivpflegenden in Deutschland

**DOI:** 10.1007/s00063-021-00880-7

**Published:** 2021-11-01

**Authors:** Ann-Kathrin Hönl, Florian Jantz, Luis Möckel

**Affiliations:** HSD Hochschule Döpfer GmbH, University of Applied Sciences, Waidmarkt 3 und 9, 50676 Köln, Deutschland

**Keywords:** Muskuloskeletale Erkrankungen, Psychische Erkrankungen, Gesundheitsfachberufe, Medikamenteneinnahme, Prävention, Musculoskeletal disorders, Mental disorders, Healthcare professionals, Analgesic intake, Prevention

## Abstract

**Ziel der Studie:**

Das Ziel dieser Studie war es, die Schmerzprävalenz, das Ausmaß der Schmerzmitteleinnahme sowie die mentale Gesundheit bei Intensivpflegenden zu untersuchen.

**Methoden:**

Bei dieser Studie handelte es sich um eine Befragung von Intensivpflegenden aus Deutschland. Neben selbstentwickelten Items wurde die Kurzversion der Depressions-Angst-Stress-Skala (DASS-21) abgefragt.

**Ergebnisse:**

Insgesamt wurden 432 Intensivpflegende (82,87 % Frauen) in die Analyse eingeschlossen. 36,81 % (95 %-Konfidenzintervall [95 %-KI] 31,31 %; 42,99 %) der Teilnehmenden gaben an, unter wiederkehrenden Schmerzen zu leiden, und 18,52 % (95 %-KI 14,68 %; 23,05 %) gaben an, unter chronischen Schmerzen zu leiden. Signifikant mit chronischen Schmerzen assoziiert waren das Körpergewicht (adjustierte Odds Ratio [AOR]: 1,02 [95 %-KI 1,01; 1,03]; *p* = 0,006) und das Geschlecht (Männer AOR: 0,33 [95 %-KI 0,14; 0,78]; *p* = 0,012) sowie mit wiederkehrenden Schmerzen das Geschlecht (Männer AOR: 0,59 [95 %-KI 0,31; 1,00]; *p* = 0,049) und das Vorliegen von Schlafproblemen (AOR: 2,16 [95 %-KI 1,41; 3,31]; *p* ≤ 0,001). Schmerzmittel wurden von 52,61 % der Studienteilnehmenden mit chronischen/wiederkehrenden Schmerzen eingenommen (häufigster Wirkstoff: Ibuprofen [81,67 %]). Teilnehmende mit chronischen wie auch wiederkehrenden Schmerzen zeigten signifikant höhere Depressions‑, Angst- und Stresslevel im Vergleich zu denen ohne Schmerzen.

**Schlussfolgerung:**

Diese Studie zeigt, dass ein großer Anteil der teilnehmenden Intensivpflegekräfte unter chronischen und wiederkehrenden Schmerzen leidet und diese mit verschiedenen Faktoren assoziiert sind.

## Abstract (en)

**Background and Aim:**

Since nurses in general are frequently affected by pain and depressive episodes, the aim of this study was to analyze the prevalence of pain, frequency of analgesic intake and the mental health of intensive care nurses.

**Methods:**

This study was a survey study of German intensive care nurses and the questionnaire comprised self-developed items and the short version of the Depression Anxiety Stress Scale (DASS-21).

**Results:**

A total of 432 intensive care nurses (82.97% female) were included in the analysis. Recurrent pain was reported by 36.81% (95% confidence interval [CI]: 31.31%; 42.99%) of study participants and chronic pain by 18.52% (95% CI: 14.68%; 23.05%). Body weight (adjusted odds ratio (AOR): 1.02 [95% CI: 1.01; 1.03]; *p* = 0.006) and sex (male AOR: 0.33 [95% CI: 0.14; 0.78]; *p* = 0.012) were significantly associated with chronic pain, and sex (male AOR: 0.59 [95% CI: 0.31; 1.00]; *p* = 0.049) and the presence of sleeping problems (AOR: 2.16 [95% CI: 1.41; 3.31]; *p* ≤ 0.001) with recurrent pain. Analgesics were used by 52.61% of study participants with chronic/recurrent pain (most frequently used substance: ibuprofen [81.67%]). Study participants with chronic and recurrent pain indicated significantly higher depression, anxiety and stress levels compared to those without pain.

**Conclusion:**

This study indicates that a large number of participating intensive care nurses are suffering from chronic and recurrent pain. Pain in this population was associated with a variety of factors.

## Einleitung

Pflegekräfte machen im deutschen Gesundheitssystem einen großen Anteil der Beschäftigten aus. So waren in 2019 rund 457.947 Pflegekräfte in Krankenhäusern und 490.110 in stationären Pflegeeinrichtungen in einem sozialversicherungspflichtigen Arbeitsverhältnis angestellt [21, 22]. Gleichzeitig zeigt der Gesundheitsreport der Techniker Krankenkasse [24], dass der Krankheitsausfall von Pflegekräften aufgrund von psychischen Diagnosen bei durchschnittlich 463 und bei den Muskel-Skelett-Erkrankungen bei 479 Arbeitsfähigkeit(AU)-Tagen pro 100 Versicherungsjahren liegt. Im Gegensatz dazu kommt die Gesamtgruppe aller Berufstätigen nur auf 247 AU-Tage bei den psychischen Erkrankungen und 261 bei den Muskel-Skelett-Erkrankungen. Zu erwähnen ist noch, dass unter den häufigsten Diagnosen bei Pflegekräften Rückenschmerzen (140 AU-Tage/100 Versicherungsjahre) und depressive Episoden (123 AU-Tage/100 Versicherungsjahre) genannt wurden. Der Report der Techniker Krankenkasse vermutet außerdem, dass es aufgrund des hohen „Verordnungsvolumens an Präparaten zur Behandlung des peptischen Ulkus“ (A02B) auch einen relativ hohen Schmerzmittelkonsum bei Pflegekräfte geben könnte (Techniker Krankenkasse, [24]). Diese Ergebnisse waren allerdings auf alle Pflegebereiche insgesamt bezogen.

Dass eine Assoziation zwischen Schmerzen und mentalen Gesundheitsproblemen existiert, ist bereits bekannt [16, 17]. So korrelieren beispielsweise Schmerzen und Depression eng miteinander, was die dafür verantwortlichen Hirnregionen sowie das neurologische System betrifft. Des Weiteren ist bekannt, dass das gemeinsame Auftreten von Schmerzen und Depressionen die Schweregrade beider Erkrankungen verstärken kann [17].

Es existieren unseres Wissens nach bisher jedoch noch keine spezifischen Daten im Kontext zur stationären Intensivpflege. Nicht zuletzt wegen des demografischen Wandels und eines immer größer werdenden Anteils von Personen im Alter von 67 Jahren oder älter [20] könnte die Zahl kritisch kranker Patienten auf deutschen Intensivstationen stetig ansteigen. Gleichzeitig ist bekannt, dass der Schweregrad von Patienten einen Einfluss auf die Arbeitsbelastung von Intensivpflegenden hat [9] und psychosoziale Bedingungen sowie physische Faktoren am Arbeitsplatz beispielsweise im Zusammenhang mit Rückenschmerzen stehen [16].

## Ziele dieser Arbeit

Da Pflegende häufiger an psychischen und Muskel-Skelett-Erkrankungen als die Gesamtgruppe der Berufstätigen leiden [24] und unseres Wissens nach explizit für die Intensivpflegenden aus Deutschland bisher keine Studien zur Thematik Schmerzen und mentale Gesundheit vorliegen, waren die Ziele dieser Arbeit

1. die Prävalenz von Schmerzen nach Art (chronisch, wiederkehrend),
2. das Ausmaß der Schmerzmitteleinnahme und
3. die mentale Gesundheit bei Intensivpflegenden zu erheben.
4. Außerdem sollten Faktoren identifiziert werden, welche mit dem Auftreten von Schmerzen bei Intensivpflegenden assoziiert sind.

## Methoden

### Studiendesign und Studienteilnehmende

Diese Studie war eine Befragung von Intensivpflegekräften in Deutschland, welche zwischen dem 24.11.2020 und dem 25.01.2021 durchgeführt wurde. Eingeschlossen wurden aktuell als Intensivpflegekräfte tätige Personen im Alter von 18 Jahren oder älter. Die Befragung wurde online mithilfe des Programms SoSci Survey (Sosci Survey GmbH, München, Deutschland) durchgeführt [18], da Webbefragungen bei sensiblen Fragestellungen von Vorteil sind und Effekte der sozialen Erwünschtheit minimiert werden [23].

In der Zeit vom 17.11.2020 bis 23.11.2020 wurde zunächst ein online Prätest mit Intensivpflegenden als auch Nichtintensivpflegenden durchgeführt. Dabei wurde der Fragebogen auf logischen Aufbau, Verständnis der Fragen, technische Funktionsfähigkeit und Dauer der Befragung getestet. Wenn nötig wurden Fragen entsprechend des Prätestfeedbacks angepasst.

Der eigentliche Link zur Onlinebefragung wurde über Gruppierungen (*Krankenschwestern und Krankenpfleger, Intensivpflege und Anästhesiepflege, Die Pflege – Krankenschwester & Krankenpfleger, Krankenschwestern und Krankenpfleger in Berlin, Intensivpflege, Wir sind die Pflege*) verteilt, in welchen Intensivpflegekräfte organisiert sind, sowie über das Schneeballprinzip. Zur Verteilung mittels Schneeballprinzip wurden die Teilnehmenden gebeten den Link in ihrem persönlichen Netzwerk aus Intensivpflegenden zu verteilen. Die Befragung wurde mit Ausnahme von Bereitstellung des Teilnahmelinks nicht weiter beworben und die Zielpopulation erhielt keine Anreize zur Teilnahme an der Studie.

Die Teilnahme an der Studie war freiwillig, anonym, konnte durch die Teilnehmer jederzeit beendet werden und erfolgte im Rahmen des geltenden Datenschutzes. Des Weiteren wurde eine nichtvulnerable Gruppe befragt und somit war Ethikvotum für die Durchführung dieser Studie nicht notwendig. Alle Teilnehmenden erteilten ihre informierte Einwilligung. Die Durchführung dieser Studie erfolgte in Anlehnung an das CHERRIES-Statement [4].

### Fragebogen

Zu Beginn der Befragung wurden im Einleitungstext Angaben zum Ziel der Studie, den Durchführenden sowie zur Weiterverarbeitung, Nutzung und Speicherung der erhobenen Daten gemacht. Des Weiteren wurde den Studienteilnehmenden die ungefähre Dauer der Befragung mitgeteilt sowie die Aufforderungen, nur einmal an dieser Studie teilzunehmen und nur dann teilzunehmen, wenn diese Intensivpflegende sind.

Der eigentliche Fragebogen bestand aus soziodemografischen (u. a. Altersgruppe, Geschlecht, Familienstand, Raucher), berufsbezogenen (u. a. Schichtdienst, Jahre Berufserfahrung, Stellenumfang) Fragen sowie aus Fragen zu Gesundheit und Gesundheitsstörungen (u. a. zu Schmerzen, Schlafstörungen, mentaler Gesundheit, Gewicht). Zum Thema Schmerzen wurden zunächst alle Studienteilnehmer gebeten, die Frage „Haben Sie momentan Schmerzen?“ mit den Antwortmöglichkeiten „Nein“, „Ja, akute Schmerzen“, „Ja, chronische Schmerzen (definiert als Schmerzen für 3 Monate oder länger [1, 7, 13])“ oder „Ja, wiederkehrende Schmerzen (definiert als wiederholt aufkommende Schmerzen für mindestens 24 h mit mindestens einem Monat Schmerz-freier Zeit dazwischen [19, 25])“ zu beantworten. Wurde das Vorliegen von Schmerzen angegeben, so sollten diese Teilnehmenden die Stärke ihrer Schmerzen anhand einer numerischen Rating-Skala bewerten (0 gleichbedeutend mit keinen Schmerzen, 10 gleichbedeutend mit den stärksten vorstellbaren Schmerzen). Des Weiteren sollten die Teilnehmenden die Schmerzlokalisation(en) anhand einer vorgegebenen Liste angeben und ob Schmerzmittel gegen diese eingenommen werden. Teilnehmende mit Schmerzmitteleinnahme wurden außerdem nach Einnahmehäufigkeit und den spezifischen Substanzen gefragt.

Alle Studienteilnehmende wurden gebeten, die deutschsprachige Kurzversion der validierten Depressions-Angst-Stress-Skala (DASS-21) zu beantworten, welche aus 21 Items besteht [6, 10, 11, 14]. Der Fragebogen enthält die Subskalen Depression, Angst und Stress, die jeweils aus 7 Items aufgebaut sind. Zur Beantwortung der einzelnen Items mussten die Studienteilnehmenden die Aussage jedes Items mit 0 (traf gar nicht auf mich zu), 1 (traf bis zu einem gewissen Grad auf mich zu oder manchmal), 2 (traf in beträchtlichem Maße auf mich zu oder ziemlich oft) oder 3 (traf sehr stark auf mich zu oder die meiste Zeit) bewerten [14].

Insgesamt wurden 45 Items für diese Analyse verwendet. Um Effekte durch die Antwortreihenfolge zu minimieren, wurde bei den selbst entwickelten Items das Forced-Choice-Antwortformat verwendet (Ausnahme Lokalisation der Schmerzen), die Antwortlisten so kurz wie möglich gehalten und die Fragen einfach, ohne abstrakte Begriffe formuliert [2]. Die Items waren in thematischen Blöcken (Soziodemografie, arbeitsbezogene Faktoren, Schmerzen, Schmerzmittel, Schlaf, Gewicht, DASS-21) eingeteilt und wurden nicht randomisiert dargestellt. Die Teilnehmenden hatten nicht die Möglichkeit, auf vorherige Seiten zu Wechseln oder den Fragebogen zum Abschluss noch einmal zu begutachten und Antworten zu ändern.

### Statistische Auswertung

Fragebögen wurden in die Analyse eingeschlossen, wenn 100 % der soziodemografischen und berufsbezogenen Fragen sowie die Frage zu vorliegenden Schmerzen beantwortet wurden. Zur Darstellung der Charakteristika der Studienteilnehmenden wurden die prozentualen Anteile entsprechender Gruppen berechnet. Zunächst wurde die Punktprävalenz von Schmerzen nach Art (akut, chronisch, wiederkehrend) sowie die dazugehörigen 95 %-Konfidenzintervalle (95 %-KI) berechnet. Anschließend wurden univariable Analysen mittels logistischer Regression, Pearson-Chi-Quadrat-Test oder Fischer’s Exact Test, unter Berechnung der Odds Ratio (OR) und dazugehöriger 95 %-KI, angewendet, um Subgruppen mit einem Risiko für chronische bzw. wiederkehrende Schmerzen zu identifizieren. Alle Variablen, welche in den univariablen Analysen mit einem *p*-Wert von *p* ≤ 0,2 mit Schmerzen assoziiert waren, wurden in eine multivariable logistische Regression eingeschlossen und unter Berechnung der adjustierten OR (AOR) und des dazugehörigen 95 %-KI analysiert.

Die Analyse der DASS-21-Subskalen erfolgte durch Aufsummieren der entsprechenden Items und anschließender Multiplikation mit dem Faktor 2, um zur Vollversion, dem DASS, äquivalente Subskalenscores zu erhalten [6, 10, 11, 14]. ANOVA mit Post-hoc-Analyse und Tukey-Korrektur wurde angewandt, um die verdoppelten DASS-21-Subskalen-Scores zwischen Studienteilnehmenden ohne, mit chronischen sowie mit wiederkehrenden Schmerzen zu vergleichen. Für die Darstellung metrischer Daten, wie der DASS-21-Subskalen oder der Einnahmehäufigkeit von Schmerzmitteln, wurden Mittelwerte und Standardfehler (SE) berechnet.

Die Teilnehmenden mit akuten Schmerzen (*n* = 6) sowie Subgruppen mit weniger als 10 Teilnehmenden wurden nicht separat analysiert. Die statistische Analyse wurde mithilfe des Programms JASP (JASP Team, University of Amsterdam, Niederlande) durchgeführt [8]. Ein *p*-Wert von ≤ 0,05 wurde für alle Analysen als statistisch signifikant betrachtet.

## Ergebnisse

### Charakteristika der Studienteilnehmenden

Insgesamt wurde die Befragung 1163-mal von Intensivpflegekräften angeklickt und 490 (42,13 %) nahmen an der Studie teil. Von diesen erfüllten 432 (37,15 %) die Einschlusskriterien und konnten in die finale Analyse eingeschlossen werden.

Von den eingeschlossenen Studienteilnehmenden (*n* = 432; Tab. 1) waren insgesamt 82,87 % Frauen und die Altersgruppen 20–29 Jahre (32,64 %), 30–39 Jahre (32,18 %) sowie 40–49 Jahre (22,92 %) waren am häufigsten Vertreten. Die Mehrzahl der eingeschlossenen Teilnehmenden lebte entweder in Partnerschaft (31,94 %), zum Beispiel als eingetragene Lebenspartnerschaft oder eheähnliche Gemeinschaft ohne Kinder, oder war verheiratet ohne Kinder (28,24 %) und 62,50 % waren Nichtraucher. Insgesamt gaben 29,86 % der eingeschlossenen Studienteilnehmenden 1–3 Jahre Berufserfahrung, 22,22 % 4–6 Jahre sowie 19,68 % mehr als 15 Jahre Berufserfahrung an. Der überwiegende Anteil von 77,79 % arbeitete in dem Schichtdienstmodell Früh-/Spät-/Nachtschicht und 57,64 % arbeiteten in Vollzeit sowie weitere 26,39 % in Teilzeit mit einem Stellenumfang von 75 bis 99 %. Alle Details zu den Charakteristika der eingeschlossenen Studienteilnehmenden sind in Tab. 1 aufgelistet.

**Table 1:** 

Charakteristika	Gesamt *N* = 432
	%/absolut
*Anteil Frauen*	82,87/358
*Alter*	
20–29 Jahre	32,64/141
30–39 Jahre	32,18/139
40–49 Jahre	22,92/99
50–50 Jahre	11,11/48
≥ 60 Jahre	1,16/5
*Art der Lebensgemeinschaft*	
Single/alleinlebend	24,54/106
In Partnerschaft lebend (nicht verheiratet, ohne Kinder)	31,94/138
Verheiratet ohne Kinder	28,24/122
Familie^a^	15,05/65
Verwitwet	0,23/1
*Raucher*^b^	
Nein	62,50/270
Ja, regelmäßig	27,78/120
Ja, gelegentlich	9,72/42
*Berufserfahrung*	
1–3 Jahre	29,86/129
4–6 Jahre	22,22/96
7–10 Jahre	14,82/64
11–15 Jahre	13,43/58
> 15 Jahre	19,68/85
*Stellenumfang*	
Vollzeit 100 %	57,64/249
Teilzeit 75–99 %	26,39/114
Teilzeit 50–74 %	12,04/52
Teilzeit ≤ 50 %	3,24/14
450 € Basis	0,69/3
*Varianten Schichtdienste*	
Früh‑, Spät- und Nachtschicht	77,79/336
Nur Früh‑/Spätschicht	9,95/43
Früh- oder Spät- oder Nachtschicht	4,63/20
Andere Variante	7,64/33

^a^Familie definiert als eheliche oder uneheliche Partnerschaft mit mindestens einem Kind im Haushalt

^b^Nein: momentan kein Raucher; regelmäßig: täglich mehrere Zigaretten; gelegentlich: max. einmal wöchentlich

### Schmerzpunktprävalenz und Risikogruppen

**Figure 1:**
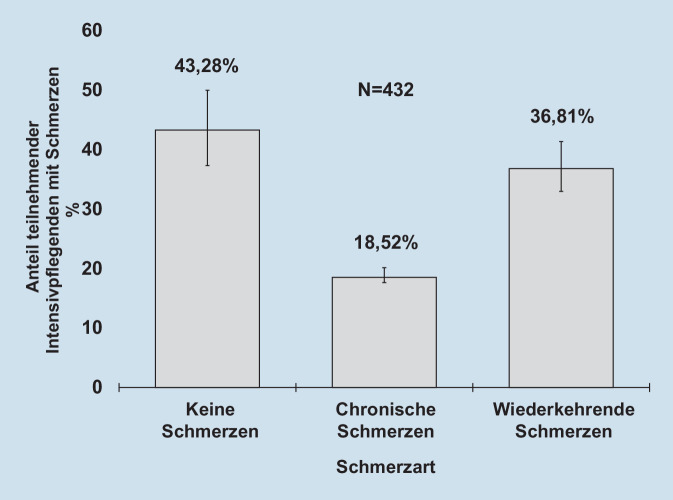

Insgesamt gaben 56,72 % (95 %-KI 49,83 %; 64,28 %; 245 von 432) der teilnehmenden Intensivpflegekräfte an, unter Schmerzen (jeglicher Art) zu leiden, wobei 36,81 % (95 %-KI 31,31 %; 42,99 %) wiederkehrende und 18,52 % (95 %-KI 14,68 %; 23,05 %) chronische Schmerzen angegeben haben (Abb. 1). Bei Studienteilnehmenden mit chronischen bzw. wiederkehrenden Schmerzen (*n* = 230) waren am häufigsten die Lendenwirbelsäule (58,70 %), der Kopf (56,52 %), die Halswirbelsäule (43,91 %), die unteren Extremitäten (23,04 %), die Brustwirbelsäule (20,00 %), der gastrointestinale Bereich (19,57 %) sowie die oberen Extremitäten (16,96 %) betroffen. Des Weiteren gaben 30,44 % eine Schmerzstärke von 2 bis 3 an, 45,65 % eine Stärke von 4 bis 5 sowie weitere 20,87 % einen Wert von 6 bis 7. Sehr starke Schmerzen von 8 bis 9 gaben 2,61 % und extreme Schmerzen von 10 gaben 0,44 % der Studienteilnehmenden mit Schmerzen an.

In der multivariablen Analyse (Tab. 2) waren das Geschlecht (Männer AOR: 0,33 [95 %-KI 0,14; 0,78]; *p* = 0,012) sowie das Körpergewicht (AOR: 1,02 [1,01; 1,03]; *p* = 0,006) mit chronischen Schmerzen assoziiert. Mit wiederkehrenden Schmerzen waren in der multivariablen Analyse ebenfalls das Geschlecht (Männer AOR: 0,59 [95 %-KI 0,31; 1,00]; *p* = 0,049) sowie insbesondere das Vorliegen von Schlafproblemen (AOR: 2,16 [95 %-KI 1,41; 3,31]; *p* ≤ 0,001) assoziiert.

**Table 2:** 

Variablen und Subgruppen	Chronische Schmerzen OR (95 %-KI)	Chronische Schmerzen AOR (95 %-KI)^a^	Wiederkehrende Schmerzen OR (95 %-KI)	Wiederkehrende Schmerzen AOR (95 %-KI)^a^
Frauen (*n* = 358)	Referenz	Referenz	Referenz	Referenz
Männer (*n* = 74)	0,48 (0,22; 1,05); *p* = 0,061	*0,33 (0,14; 0,78); p* *=* *0,012*	0,58 (0,33; 1,02); *p* = 0,055	*0,59 (0,31; 1,00); p* *=* *0,049*
**Alter**				
20–29 Jahre (*n* = 141)	Referenz	Referenz	Referenz	Referenz
30–39 Jahre (*n* = 139)	1,52 (0,79; 2,98); *p* = 0,218	1,24 (0,54; 2,85); *p* = 0,610	0,93 (0,56; 1,53); *p* = 0,767	1,08 (0,60; 1,92); *p* = 0,799
40–49 Jahre (*n* = 99)	*2,08 (1,04; 4,17); p* *=* *0,036*	1,36 (0,50; 3,66); *p* = 0,549	1,49 (0,88; 2,52); *p* = 0,14	1,62 (0,80; 3,29); *p* = 0,795
50–59 Jahre (*n* = 48)	*3,65 (1,66; 8,00); p* *≤* *0,001*	1,84 (0,56; 6,01); *p* = 0,314	1,38 (0,71; 2,71); *p* = 0,342	1,08 (0,42; 2,81); *p* = 0,872
≥ 60 Jahre (*n* = 5)	–	–	–	–
Körpergewicht – pro kg Anstieg	*1,02 (1,00; 1,03); p* *=* *0,011*	*1,02 (1,01; 1,03); p* *=* *0,006*	1,00 (0,99; 1,01); *p* = 0,837	–
**Art der Lebensgemeinschaft**				
Single/alleinlebend (*n* = 106)	Referenz	–	Referenz	–
In Partnerschaft lebend (nicht verheiratet, ohne Kinder) (*n* = 138)	0,90 (0,47; 1,71); *p* = 0,737	–	1,23 (0,72; 2,09); *p* = 0,451	–
Verheiratet ohne Kinder (*n* = 122)	1,04 (0,54; 2,00); *p* = 0,898	–	1,36 (0,79; 2,34); *p* = 0,265	–
Familie^b^ (*n* = 65)	0,65 (0,28; 1,52); *p* = 0,319	–	1,04 (0,54; 2,00); *p* = 0,911	–
Verwitwet (*n* = 1)	–	–	–	–
**Raucher**				
Nein (*n* = 270)	Referenz	Referenz	Referenz	Referenz
Ja, regelmäßig (*n* = 120)	1,52 (0,89; 2,59); *p* = 0,119	1,62 (0,89; 2,94); *p* = 0,112	0,86 (0,55; 1,34); *p* = 0,526	0,91 (0,56; 1,46); *p* = 0,694
Ja, gelegentlich (*n* = 42)	1,00 (0,42; 2,39); *p* = 1,00	1,13 (0,43; 2,93); *p* = 0,810	1,57 (0,82; 3,02); *p* = 0,174	1,57 (0,80; 3,08); *p* = 0,193
**Schlafprobleme**				
Nein (*n* = 183)	Referenz	–	Referenz	Referenz
Ja (*n* = 249)	1,12 (0,69; 1,86); *p* = 0,636	–	*2,17 (1,43; 3,23); p* *≤* *0,001*	*2,16 (1,41; 3,31); p* *≤* *0,001*
**Berufserfahrung**				
1–3 Jahre (*n* = 129)	Referenz	Referenz	Referenz	Referenz
4–6 Jahre (*n* = 96)	0,94 (0,43; 2,08); *p* = 0,881	0,68 (0,29; 1,60); *p* = 0,381	1,30 (0,75; 2,25); *p* = 0,352	1,33 (0,75; 2,36); *p* = 0,327
7–10 Jahre (*n* = 64)	1,22 (0,52; 2,84); *p* = 0,645	1,13 (0,42; 3,02); *p* = 0,812	1,16 (0,62; 2,18); *p* = 0,64	1,08 (0,53; 2,21); *p* = 0,828
11–15 Jahre (*n* = 58)	2,50 (1,16; 5,42); *p* = 0,017	1,73 (0,66; 4,57); *p* = 0,267	0,86 (0,44; 1,69); *p* = 0,658	0,67 (0,30; 1,47); *p* = 0,301
≥ 15 Jahre (*n* = 85)	2,75 (1,37; 5,48); *p* = 0,003	1,91 (0,67; 5,49); *p* = 0,228	1,84 (1,05; 3,23); *p* = 0,033	1,44 (0,63; 3,28); *p* = 0,390
**Stellenumfang**				
Vollzeit 100% (*n* = 249)	Referenz	Referenz	Referenz	–
Teilzeit 75–99% (*n* = 114)	1,28 (0,73; 2,23); *p* = 0,388	0,86 (0,45; 1,66); *p* = 0,660	1,15 (0,73; 1,82); *p* = 0,55	–
Teilzeit 50–74% (*n* = 52)	0,75 (0,31; 1,76); *p* = 0,502	0,43 (0,16; 1,15); *p* = 0,093	1,45 (0,79; 2,66); *p* = 0,227	–
Teilzeit ≤ 50% (*n* = 14)	*3,59 (1,19; 10,88); p* *=* *0,017*	1,94 (0,58; 6,51); *p* = 0,281	0,50 (0,09; 1,96); *p* = 0,392	–
450 €-Basis (*n* = 3)	–	–	–	–
**Schichtdienste**				
Früh‑, Spät- und Nachtschicht (*n* = 336)	Referenz	Referenz	Referenz	–
Früh- und Spätschicht (*n* = 43)	*2,37 (1,16; 4,84); p* *=* *0,016*	2,19 (0,98; 4,91); *p* = 0,056	0,65 (0,32; 1,30); *p* = 0,218	–
Früh- oder Spät- oder Nachtschicht (*n* = 20)	*3,62 (1,22; 10,20); p* *=* *0,004*	2,71 (0,94; 7,80); *p* = 0,066	1,36 (0,55; 3,38); *p* = 0,502	–
Andere Variante (*n* = 33)	1,47 (0,61; 3,56); *p* = 0,391	1,23 (0,47; 3,27); *p* = 0,675	0,95 (0,45; 2,00); *p* = 0,898	–

^a^Eingeschlossen wurden alle Variablen mit *p* ≤ 0,2 (mindestens eine Subgruppe) in den univariablen Analysen

^b^Familie definiert als eheliche oder uneheliche Partnerschaft mit mindestens einem Kind im Haushalt

### Schmerzmitteleinnahme und mentale Gesundheit

**Figure 2:**
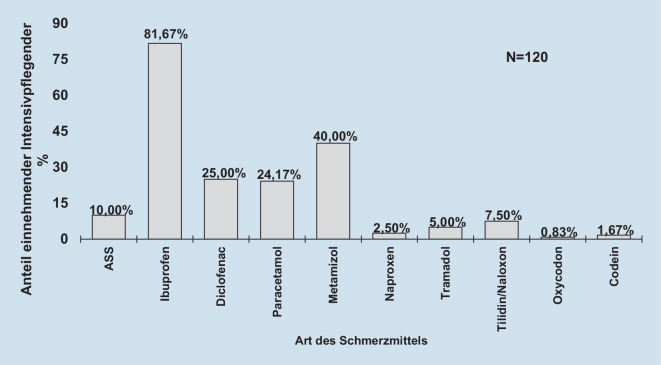

Insgesamt gaben 52,61 % (121 von 230) der teilnehmenden Intensivpflegekräfte mit chronischen bzw. wiederkehrenden Schmerzen an, dass sie Schmerzmittel einnehmen, wobei die Einnahmehäufigkeit mit durchschnittlich 2,93 (SE 0,31; *n* = 78) pro Woche angegeben wurde. Die Einnahmewahrscheinlichkeit war unter denjenigen mit chronischen Schmerzen (58,23 %) nicht signifikant höher (OR 1,41 [95 %-KI 0,82; 2,45]; *p* = 0,217) als bei denen mit wiederkehrenden Schmerzen (49,67 %). Die häufigsten eingenommenen Schmerzmittel (≥ 2 %; beide Schmerzarten zusammen) waren Ibuprofen (81,67 %), Metamizol (40,00 %), Diclofenac (25,00 %), Paracetamol (24,17 %), Acetylsalicylsäure (10,00 %), Tilidin/Naloxon (7,50 %), Tramadol (5,00 %) und Naproxen (2,50 %; Abb. 2).

**Figure 3:**
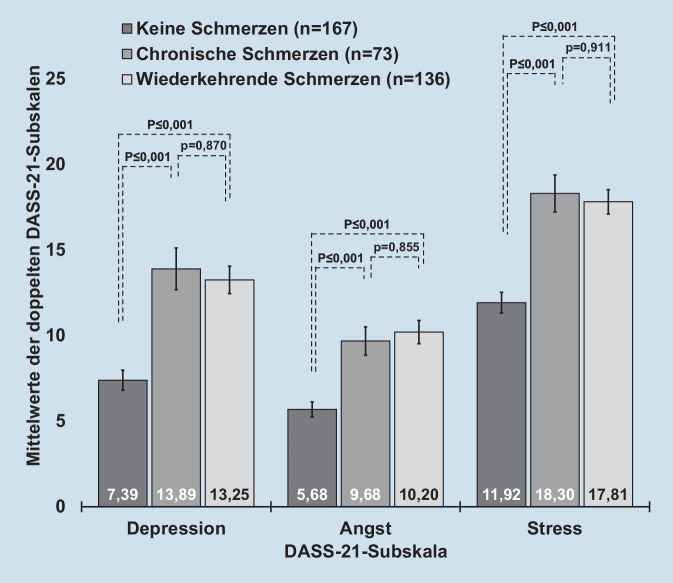

Insgesamt zeigte die Gesamtpopulation der Studienteilnehmenden (*n* = 387) doppelte DASS-21-Scores von 10,86 (SE 0,48) für Depression, 8,21 (SE 0,37) für Angst und 15,40 (SE 0,45) für Stress. Die Studienteilnehmenden mit chronischen als auch wiederkehrenden Schmerzen zeigten einen signifikant höheren Depressions‑, Angst- und Stressscore (jeweils *p* ≤ 0,001) im Vergleich zu Teilnehmenden ohne Schmerzen (Abb. 3). Dabei war bei chronischen/wiederkehrenden Schmerzen der Depressionsscore um 79–88 %, der Angstscore um 70–80 % sowie der Stressscore um 49–54 % im Vergleich zu denen ohne Schmerzen erhöht.

## Diskussion

Diese Studie zeigt, dass etwa 56 % der teilnehmenden Intensivpflegekräfte an chronischen bzw. wiederkehrenden Schmerzen litten, wobei fast jeder fünfte Intensivpflegende chronische Schmerzen angab. Diese Schmerzprävalenz ist vergleichbar mit der bei deutschen Rettungskräften, welche in einer Studie mit etwa 59 % angegeben wurde. Allerdings ist die Prävalenz chronischer Schmerzen bei den teilnehmenden Intensivpflegekräften mit 19 % höher im Vergleich zu den Rettungskräften mit 11 % [13]. Internationale Studien gaben für Pflegende Schmerzprävalenzen von 70 bis 82 % an, wobei es sich hierbei um 12-Monats-Prävalenzen handelte [3, 5, 26], wohingegen in der vorliegenden Studie die Schmerzen am Tag der Teilnahme abgefragt wurden und der Zeitraum zwei Monate betrug. In der Studie von Freimann et al., mit Pflegenden aus Estland, wurde allerdings auch die Schmerzprävalenz im letzten Monat mit 64 % angegeben [5], was sicherlich vergleichbar mit den 56 % in der vorliegenden Studie ist.

Von den Teilnehmenden mit Schmerzen gaben 58,70 % Schmerzen im Bereich der LWS und 43,91 % Schmerzen im Bereich der HWS an. Diese Ergebnisse verifizieren damit die Daten der Techniker Krankenkasse, welche Rückenschmerzen bei Pflegekräften im Allgemeinen unter den drei häufigsten Diagnosen führt [24], und transferieren die Problematik von Rückenschmerzen somit auch auf den Bereich der Intensivpflege.

Faktoren, welche in der multivariablen Analyse bei den teilnehmenden Intensivpflegekräften signifikant mit Schmerzen assoziiert waren, waren das Köpergewicht, das Geschlecht sowie das Vorliegen von Schlafproblemen. Offen bleibt sicherlich ob die Schlafprobleme zu den Schmerzen geführt haben oder Schmerzen aufgrund der Schlafprobleme auftreten, dafür sind weitere Untersuchungen notwendig. Im Gegensatz zur vorliegenden Studie zeigten in einer Arbeit von Mekonnen auch das Arbeiten im Schichtdienst, die Arbeitserfahrung sowie die Tatsache, ob man ein Sicherheitstraining erhalten hat, eine signifikante Assoziation mit dem Auftreten von Schmerzen [12].

Etwa 53 % der Teilnehmenden mit chronischen bzw. wiederkehrenden Schmerzen nehmen regelmäßig Schmerzmittel ein. Metamizol (40,00 %) wird nach Ibuprofen (81,67 %) am zweithäufigsten verwendet. Dies liegt womöglich an der rezeptfreien Verfügbarkeit von Ibuprofen bis zu einer Wirkstärke von 400 mg. Auch hier werden die Vermutungen der Techniker Krankenkasse bzgl. einer gehäuften Schmerzmitteleinnahme bei Pflegekräfte im Allgemeinen [24] für die Intensivpflegenden bestätigt. Zu erwähnen ist, dass etwa 13 % der Teilnehmenden die Nutzung von Opioiden (Tilidin 7,50 %/Tramadol 5,00 %) angaben. Für die Allgemeinbevölkerung in Deutschland ist für Personen mit Kopfschmerzen bzw. Migräne bekannt, dass 46–50 % Ibuprofen, 6,0–9,3 % Metamizol, 0,7 % Tramadol und 0,5 % Tilidin einnehmen [15]. Auch wenn die Anzahl der teilnehmenden Intensivpflegenden, welche die oben genannten Medikamente einnehmen, höher erscheint als in der Allgemeinbevölkerung, lässt sich dies aufgrund der verschiedenen untersuchten Schmerzarten und -lokalisationen in dieser Studie nicht final schlussfolgern.

Als bedenklich zu nennen ist sicherlich, dass die teilnehmenden Intensivpflegekräfte mit chronischen bzw. wiederkehrenden Schmerzen signifikant höhere Depressions‑, Angst- und Stressscores (Abb. 3) aufwiesen. Offen bleibt die Frage, ob die mentale Gesundheit aufgrund der Schmerzen schlechter ist oder Schmerzen aufgrund einer schlechteren mentalen Gesundheit auftreten. Ein Zusammenhang zwischen der Psyche und Schmerzen ist bereits bekannt. So werden die Depression und das Stressempfinden als sogenannte „yellow flags“ (psychosoziale Risikofaktoren) bezeichnet, welche das Risiko für den Übergang von einem akuten in einen chronischen Verlauf bezogen auf Rückenschmerzen erhöhen können [16]. Gerade das Thema Schmerzen und mentale Gesundheit sollte in weiteren Studien untersucht werden. So sollte im Folgenden über validierte Fragebögen zu operativen und organisationsspezifischen Stressoren diejenigen Faktoren identifiziert werden, welche im beruflichen Alltag von Intensivpflegenden sowohl die mentale Gesundheit als auch das Auftreten von Schmerzen negativ beeinflussen.

Diese Studie hat mehrere Limitationen.

Erstens ist die Studienpopulation mit 432 Teilnehmenden sehr klein und wir können nicht abschätzen, ob die Studienpopulation der Grundgesamtheit der Intensivpflegenden in Bezug auf Geschlechts- und Altersverteilung sowie insbesondere das Vorliegen von schmerzbegünstigenden Faktoren entspricht. Außerdem besteht die Gefahr, dass vermehrt Intensivpflegende mit Schmerzen an dieser Studie teilgenommen haben, was sich in kleinen Stichproben besonders auswirken kann, sodass die Daten nur limitiert repräsentativ sind.

Zweitens wurde die Studie während der Coronapandemie durchgeführt und wir können nicht bewerten, inwiefern die Ergebnisse durch die Pandemie beeinflusst wurden.

Drittens handelt es sich um eine online durchgeführte Befragung und durch den fehlenden Kontakt zu den Teilnehmenden müssen wir uns somit auf deren Ehrlichkeit und richtiges Ausfüllen des Fragebogens verlassen sowie darauf, dass diese nicht mehrfach an der Befragung teilgenommen haben.

Viertens hat der Fragebogen selbst ebenfalls Limitationen. So wurden für die Analyse nur geschlossene Frage verwendet und es bestand nur teilweise die Möglichkeit, Sonstiges/Anderes mit Freitextfeldoption auszuwählen. Bei geschlossenen Fragen besteht die Problematik, dass die Teilnehmenden durch diese beeinflusst werden, sie vereinfachen aber gleichzeitig die statistische Auswertung.

Eine Stärke unserer Befragung ist sicherlich, dass diese online durchgeführt wurde, was Effekte der sozialen Erwünschtheit bei sensiblen Fragen zu Gesundheit und Schmerzmittelkonsum bestmöglich minimierte [23].

### Fazit

- Diese Studie zeigt, dass chronische Schmerzen bei 19 % und wiederkehrende bei 37 % der teilnehmenden Intensivpflegenden auftraten.
- Von diesen gaben außerdem etwa 53 % an, Schmerzmittel einzunehmen.
- Es konnten für die Studienteilnehmenden signifikante Assoziationen zwischen dem Auftreten von chronischen bzw. wiederkehrenden Schmerzen und dem Geschlecht, Körpergewicht sowie Schlafproblemen identifiziert werden.
- Die Daten der vorliegenden Analyse deuten zusätzlich darauf hin, dass der Gesundheitszustand von Intensivpflegenden in Deutschland als bedenklich einzustufen ist und somit präventive sowie gesundheitsförderliche Maßnahmen für diese Berufsgruppe notwendig sind.
